# Alginate and Algal-Based Beads for the Sorption of Metal Cations: Cu(II) and Pb(II)

**DOI:** 10.3390/ijms17091453

**Published:** 2016-09-01

**Authors:** Shengye Wang, Thierry Vincent, Catherine Faur, Eric Guibal

**Affiliations:** 1 Ecole des mines d’Alès, Centre des Matériaux des Mines d’Alès (C2MA), Pôle Matériaux Polymères Avancés (MPA) 6, Avenue de Clavières, Alès F-30319 Cedex, France; Shengye.Wang@mines-ales.fr (S.W.); thierry.vincent@mines-ales.fr (T.V.); 2 Institut Européen des Membranes-IEM (UMR 5635 CNRS-ENSCM-UM2)-Equipe Génie des Procédés Membranaires, Université Montpellier cc047, Place Eugene Bataillon, Montpellier 34095 Cedex 5, France; Catherine.Faur@umontpellier.fr

**Keywords:** algal beads, sorption, heavy metal

## Abstract

Alginate and algal-biomass (*Laminaria digitata*) beads were prepared by homogeneous Ca ionotropic gelation. In addition, glutaraldehyde-crosslinked poly (ethyleneimine) (PEI) was incorporated into algal beads. The three sorbents were characterized by scanning electron microscopy (SEM) coupled with energy dispersive X-ray analysis (EDX): the sorption occurs in the whole mass of the sorbents. Sorption experiments were conducted to evaluate the impact of pH, sorption isotherms, and uptake kinetics. A special attention was paid to the effect of drying (air-drying vs. freeze-drying) on the mass transfer properties. For alginate, freeze drying is required for maintaining the porosity of the hydrogel, while for algal-based sorbents the swelling of the material minimizes the impact of the drying procedure. The maximum sorption capacities observed from experiments were 415, 296 and 218 mg Pb g^−1^ and 112, 77 and 67 mg Cu g^−1^ for alginate, algal and algal/PEI beads respectively. Though the sorption capacities of algal-beads decreased slightly (compared to alginate beads), the greener and cheaper one-pot synthesis of algal beads makes this sorbent more competitive for environmental applications. PEI in algal beads decreases the sorption properties in the case of the sorption of metal cations under selected experimental conditions.

## 1. Introduction

For the last few decades, the regulations for metal content in drinking water but also concerning the permissible levels for their discharge into the environment have been seriously reinforced making the control of metal concentration in the industrial effluents a challenging issue for Industry. For Cu and Pb, the European Union directive on water quality set the permissible levels to 2 mg·L^−1^ and 10 µm·L^−1^ in drinking water, respectively [1]. There are also incentive politics for recycling the metals from spent materials (i.e., spent catalysts, wastes of electrical and electronic equipment (WEEEs), metal-containing sludge, etc.) because of the increasing demand on precious and strategic metals and the limited availability of primary resources [2,3]. As a consequence recovery of metal ions from industrial wastewaters recently became a strategic issue. For these reasons, a strong research effort has been performed for the last decades for developing alternative processes for the recovery of metals from wastewaters and/or waste leachates. Conventional processes such as precipitation, solvent extraction, membrane techniques are facing several constraints like economic competitiveness, environmental impact, technical efficiency that limit their use. Sorption processes have retained a great attention, especially for the treatment of dilute effluents, offering a good compromise between efficiency and competitiveness. A sorption process requires small-occupied space and in many cases is highly effective for removing metals to permissible limits. However, it suffers from two main drawbacks. One is the high cost of adsorbent preparation such as high temperature treatment during carbonization and activation in case of activated carbon [4]. The other is the difficulty of separation of spent sorbents from the treated water. For the first problem, low-cost and efficient materials are constantly being developed as alternative sorbents [5]. Numerous studies have advocated the utilization of marine resources as biosorbents for the removal of heavy metals from contaminated wastewater, as they are abundant, renewable and environmentally-friendly in terms of elimination at the end of their life-cycle [6]. Algal biomass [7], crustacean shells and resources from agriculture wastes [8,9] represent alternative materials that can be used for metal sorption/biosorption.

By comparing the sorption capacity of non-chemically modified green, red and brown algae on Pb(II) [10,11,12,13,14,15] and Cu (II) [13,14,15,16,17,18], brown algae appear to possess the best sorption performance (5–283 mg·g^−1^ for Pb(II) and 51–106 mg·g^−1^ for Cu(II). This higher removal performance is mainly explained by the presence of alginate (anionic polysaccharides) as one of the major components of brown algae [19], whose high affinity for heavy metals due to a high content of carboxyl acid groups has been confirmed by many studies [20,21]. Usually, a smaller size of sorbents associated with a higher specific surface area leads to a more rapid equilibrium compared to bigger particles [22], so these materials were mostly ground to the size ranging from 0.2–1.2 mm [14,23,24]. This, in turn, makes the beneficial utilization of algae limited by the difficulty in recovering and collecting these biosorbent particles after the sorption process. In addition these materials are generally difficult to use in conventional fixed-bed column systems due to head loss and clogging effects. This separation problem can be addressed by encapsulation of these powders into biopolymers such as chitosan, or alginate. These polymers have intrinsic sorption properties due to the presence of amine groups for chitosan [25] or the presence of carboxylic acid groups for alginate. Alginate, a linear polysaccharide composed of β-d-mannuronate (M) and α-l-guluronate (G), is another natural polymer extracted from brown algae, naturally containing carboxyl groups in each constituent residue [26]. In addition, the acid-base properties or their dissolving properties have been used for conditioning the materials under the form of spherical hydrogels for the encapsulation of active substances, sorbent micro-particles [27,28] and liquid extractants [29]. Alginate has been applied in a variety of industries such as pharmaceutical (conventional use) [30], food textile and increasingly used as coagulants and sorbents for heavy metal ions removal over the last decade. The carboxylate function of this polysaccharide is thought to account for its high affinity for divalent cations such as Pb(II), Cu(II), Cd(II), etc. In addition, numerous studies managed to incorporate functional material such as activated carbon to further improve its sorption capacity [31] or maghemite nanoparticles to enhance its separation ability [32]. However, these studies failed to reduce the cost of the sorbents or make the sorbent more environmentally friendly. On the other hand, a much more green and low-cost material, algal biomass, is usually ground finely and is hampered by the difficulty of solid-liquid separation after sorption process. Ca(II) has been widely applied as gelling agent [33] due to its simplicity, relative low-cost, and biocompatibility [34]. The method of dropping the mixture containing sodium alginate and biosorbents (mostly in powder form) into a CaCl_2_ solution to form alginate gel beads (with “egg-box” structure) has been extensively investigated. However, this “external gelation” (or heterogeneous gelation), where beads are obtained by an external Ca(II) source, was found to lead to heterogeneous beads with a more rigid surface layer than center layer due to the delay of Ca(II) diffusion after the formation of the surface layer [35]. In addition, the repeated cycles of sorption and desorption under different acid-base conditions frequently lead to partial dissolving of the biopolymer (which was gelled under “external gelation” conditions) and progressive loss of sorption capacity. In contrast, alginate beads obtained by the “internal gelation” method revealed more stable and homogeneous. The method consists in the progressive release of acid inside the bead, which, in turn, reacts with a calcium salt to release Ca(II) ions: these divalent cations react with carboxylic groups on biopolymer chains to form a tridimensional stable and homogeneous network [36,37]. The hydrogel beads are then more stable in successive sorption/desorption cycles.

Brown algal species contain approximately 30%–40% alginate [38], providing an internal alginate source for the synthesis of hydrogel beads, apart their intrinsic sorption properties. Thus, algal biomass can be prepared with alginate being simultaneously extracted and shaped under the form of hydrogel beads (one-pot synthesis process). This is not only a way to simplify the preparation procedure of the sorbents, but it also reduces the cost for sorbent resource by avoiding the extraction step of alginate. This is also a much greener procedure that allows reducing the use of and release of reagents and a loss of matter. Many studies have reported the encapsulation of algae powder into calcium alginate beads [39,40,41]; however, this is the first time brown algae have been conditioned under the form of hydrogel beads by a so-simple method for metal binding.

Polyethyleneimine (PEI) is well known for its metal chelating characteristic due to the presence of a large number of amine groups [42] and it is often used to modify the sorbent surface to increase its adsorption capacity [43]. In the previous studies, improvements of sorption properties for Zn(II), Cd(II) and Cu(II) in complex system (in the presence of Na(I), K(I) or Ca(II)) by glutaraldehyde-crosslinked PEI immobilized in alginate matrixes [44], and for Cu(II), Zn(II) and Ni(II) by tannic acid-grafted PEI encapsulated in alginate beads [45] have already been found. The incorporation of this high-density amine-bearing compound in alginate or algal capsules is supposed to introduce supplementary reactive groups with the objective to extend the range of pH in which the sorbent can be used, to enhance sorption capacities and improve sorption selectivity. This study is part of a wider research project involving the recovery of base and precious metals (which form chloro-anionic metal ions in HCl solutions). An ongoing work shows that glutaraldehyde-crosslinked PEI incorporated in algal biomass beads significantly contributes to a substantial increase of metal-anion binding.

Therefore, in this study, alginate and algal biomass (*Laminiaria digitata*) were used for the synthesis of bead-shaped sorbents: the materials were ionotropically gelled with Ca(II) using the so-called internal gelation method. In addition, glutaraldehyde-crosslinked polyethyleneimine (PEI) was incorporated into algal beads. Though the sorption of copper and lead by alginate beads [46,47,48] and by algal biomass [9,11,14,49] has been widely described, this is the first time algal biomass has been conditioned under the form of spherical beads for improving sorbent management and uptake performance (especially with the homogeneous ionotropic gelation method). The study of sorption properties with alginate beads (herein used a reference) allows evaluating the potential of these new materials under comparable experimental conditions. The removal performances of these three sorbents for Pb(II) and Cu(II) were compared in simple and complex systems to take into account the potential presence of coexisting ions present in the effluents such as Na(I) or Ca(II) (as an example, Ca(II) concentration may reach 50 and 1500 mg·L^−1^ in municipal and industrial wastewaters, respectively [50]). Moreover, a special attention has been paid to the effect of drying conditions (i.e., air-drying vs. freeze-drying) on the morphology of the materials (bead shrinking and swelling) and its possible effect on the mass transfer properties of the three sorbents. As part of a wider research work the general study is expected to contribute to a better understanding of the field of application of these materials (type of metals to be aimed for removal and/or decontamination; i.e., metal cations vs. metal anions). It is also interesting discussing the rationale use of alginate-based materials in algal biomass compared to pure alginate beads (on the basis of alginate content in the algal biomass). It is noteworthy that these sorbents were designed and applied for the pre-treatment of metal-bearing solutions and not for the purification of aqueous streams to reach drinkable levels.

## 2. Results and Discussion

### 2.1. SEM-EDX Analysis

**Figure 1:**
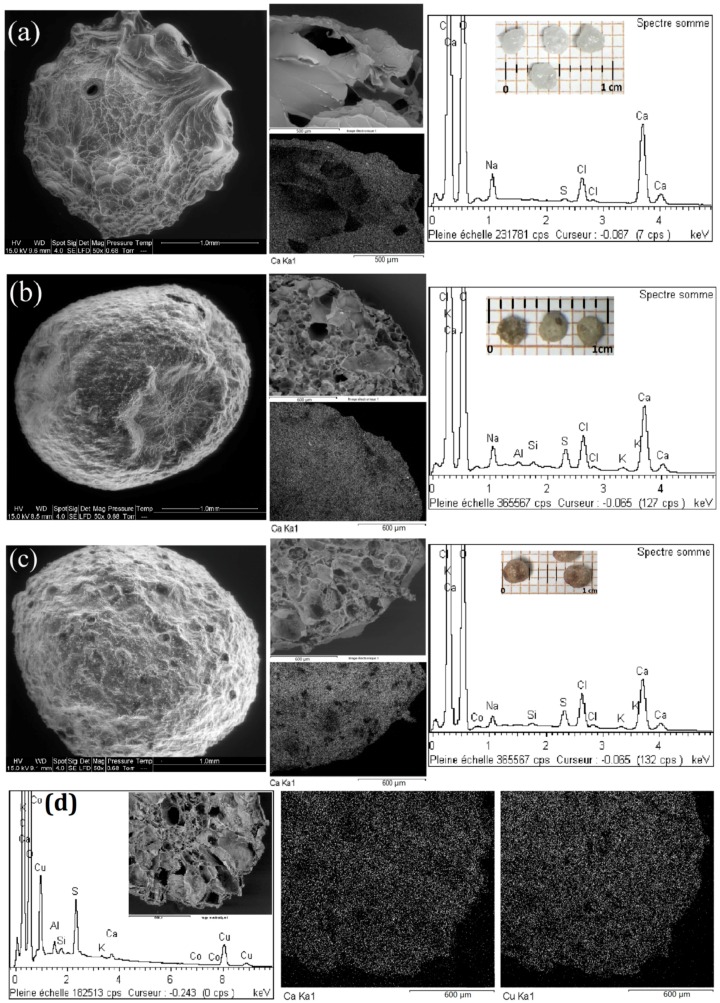
SEM-EDX micrographs of alginate beads (**a**); algal beads (**b**); algal/PEI beads (**c**); and Cu(II)-loaded algal beads (**d**).

SEM observation in Figure 1 shows the surface features of freeze-fried materials: (a) alginate beads; (b) algal beads; and (c) algal/PEI beads, at a magnification of 50.

The presence of other elements such as S in all the sorbents, K in algal-based beads and Al in algal beads were also observed. Sulfur could be associated with fucoidan residues present in the brown algae, from which alginate was extracted, and this could also explain the more intense peak of S in algal-based beads than in pure alginate beads. Light metals such as K are commonly found in untreated biomass while Al can probably be attributed to impurities associated with alginate extraction step [44]. Being superimposed with the large peaks of C and O elements, N element associated with amine groups from algal-PEI composite is not detected by EDX technique.

**Figure S1:**
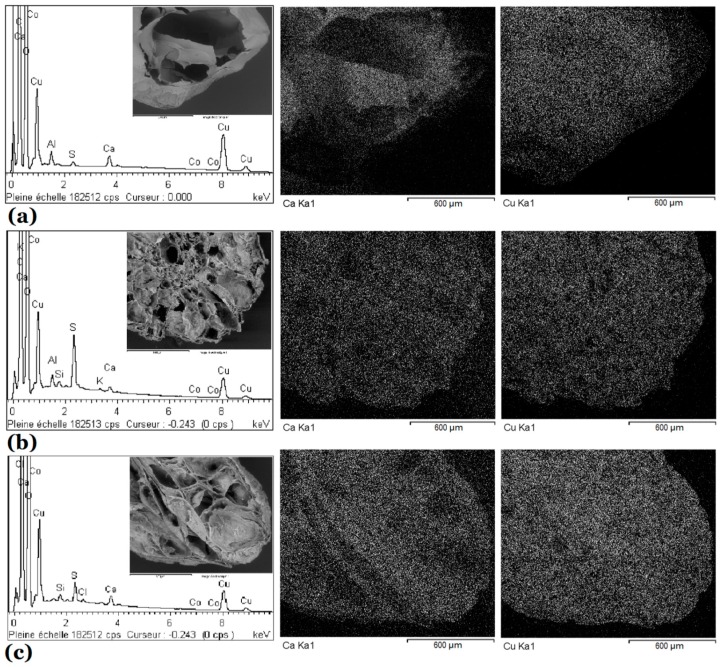
Scanning electron microscopy-energy dispersive X-ray analysis (SEM-EDX) micrographs of Cu-loaded (**a**) alginate beads; (**b**) algal beads; and (**c**) algal/glutaraldehyde-crosslinked poly (ethyleneimine) beads.

SEM micrographs of the Cu(II)-loaded algal beads shown in Figure 1d confirm that Cu(II) was sorbed and distributed homogeneously all over the entire mass of the beads and similar images were obtained for the other two Cu(II)-loaded sorbents (shown in Figure S1), suggesting that all reactive groups remain accessible and a sufficient time has been given for metal ions to migrate to the center of sorbent particles.

The internal ionotropic gelation mechanism is contributing to create more homogeneous porous structure than the conventional external gelation method. In this external gelation a very compact outer skin is formed that may contribute to reduce mass transfer properties. In the case of the internal method: the production of CO_2_ bubbles facilitates the formation of very large pores and the gelling is operating in all the directions contrary to the other method that obeys a radial process (shrinking core).

### 2.2. Effect of pH on Metal Sorption

Experiments were performed in batch under similar experimental conditions with initial pH varying between 1 and 5.5. Experimental conditions (pH and metal concentration) have been selected to avoid precipitation phenomena. Investigating the impact of pH is a key step in the design of the sorption process since this parameter may affect several critical criteria such as the speciation of the metal in the solution (in function of the presence of ligands or by hydrolysis mechanisms that may change the overall charge of metal ions in the solution), the chemical state of reactive groups on the sorbent (protonation/deprotonation), which, in turn may impact their affinity for target metal ions.

**Figure 2:**
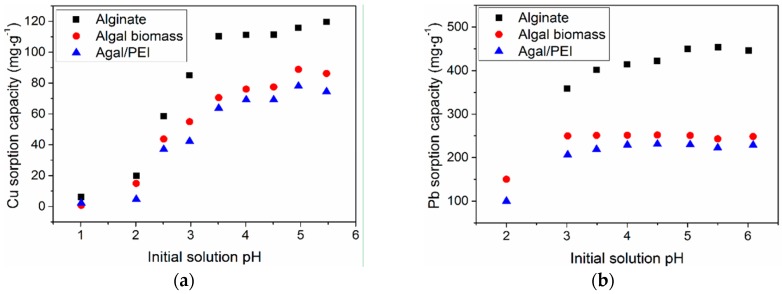
Effect of initial pH on Cu(II) (**a**) and Pb(II) (**b**) sorption (T = 25 ± 1 °C; Contact time, t = 48 h; for Cu(II): sorbent dosage, SD = 200 mg·L^−1^, *C*_0_ = 50 mg·L^−1^, for Pb(II): SD = 170 mg·L^−1^ for alginate and 375 mg·L^−1^ for algal-based sorbents, *C*_0_ = 100 mg·L^−1^).

Figure 2 clearly shows that the pH has a critical impact on the sorption of both Cu(II) and Pb(II). At low pH (below pH 2.5) sorption capacities (*q*, mg metal g^−1^) are negligible. This is obviously associated to the protonation of reactive groups at the surface of sorbent beads: the protonation of these reactive groups (amine functions for algal beads and for algal/PEI beads and carboxylic groups for the three sorbents), which, in turn have weak affinity for metal cations. As the pH increases, the protonation of the reactive groups progressively decreases and the sorption capacity significantly increases. The steep increase in the sorption capacity is essentially observed between pH 2 and 4; above pH 4 the sorption capacity tends to stabilize. The critical changes in sorption efficiency around pH 3.5 can be correlated to the acid-base properties of the encapsulating material (alginate and analogous biopolymers for algal beads). Haug [51] reported the dissociation constants of the carboxylic groups that constitute alginate biopolymer: the p*K*_a_ of carboxylic groups in mannuronic acid units is close to 3.38 while for guluronic acid the carboxylic groups have a slightly higher p*K*_a_ (i.e., close to 3.65). Below pH 3.65, carboxyl (COO–) is thus strongly associated with protons rather than with cationic metal ions (Equation (1)) and sorption remains negligible.

(1) $${\left(R–{\mathrm{COO}}^{-}\right)}_{2}–{\mathrm{Ca}}^{2+}+2H^{+}+{\mathrm{Cu}}^{2+}\to 2\left(R–\mathrm{COO}–H\right)+{\mathrm{Ca}}^{2+}+{\mathrm{Cu}}^{2+}$$

Over pH 3.38–3.65, the carboxylic groups are less protonated and then gel may form between these groups and Pb(II) or Cu(II) (Equation (2)) by ionotropic gelation [52], and Pb(II) and Cu(II) can be exchanged with Ca(II) ions.

(2) $${\left(R–{\mathrm{COO}}^{-}\right)}_{2}–{\mathrm{Ca}}^{2+}+{\mathrm{Cu}}^{2+}\to {\left(R–{\mathrm{COO}}^{-}\right)}_{2}–{\mathrm{Cu}}^{2+}+{\mathrm{Ca}}^{2+}$$

It is noteworthy that the sorption of copper appears to be slightly more affected by the pH than lead removal. Indeed, especially in the case of algal and algal/PEI beads the sorption capacity tends to form a plateau at pH above 3. The differences in sorption capacities between alginate beads and algal-based beads are directly correlated to different sorbent dosages (SD): the SD is about halved for alginate beads compared to the other sorbents; however, the sorption capacities were not doubled. This is a first indication of the interest of algal-based capsules in terms of rationale use of alginate-based fraction (the fraction of alginate in the algal biomass does not exceed 31%). In the case of algal biomass, the presence of other compounds (such as amino-acids, cellulose and other polysaccharides such as fucoidan) makes the modeling and interpretation of pH effects more complex. The amino groups may react with metal ions through different modes depending on the pH, the composition of the solution, and the speciation of the metals (more specifically their ability to form anionic species, under specific experimental conditions): metal anions in acidic solutions may be bound by electrostatic attraction/anion exchange, while metal cations will be preferentially bound to amine groups in near-neutral solutions (through interactions with the free electron doublet on nitrogen). As a consequence, in the case of Pb(II) and Cu(II) that do not form stable anionic complexes in acidic solutions (at least under selected experimental conditions) metal binding is not expected to occur on protonated amine groups and the sorption capacity is not improved due to the presence of these new functional groups. Similar conclusions can be roughly deduced from the sorption behaviors of Algal/PEI beads regarding pH effect. This also explains the fact that the effect of the pH on metal sorption is not significantly changed: sorption capacity increases with the progressive deprotonation of carboxylic groups and amine groups with pH increase.

**Figure S2:**
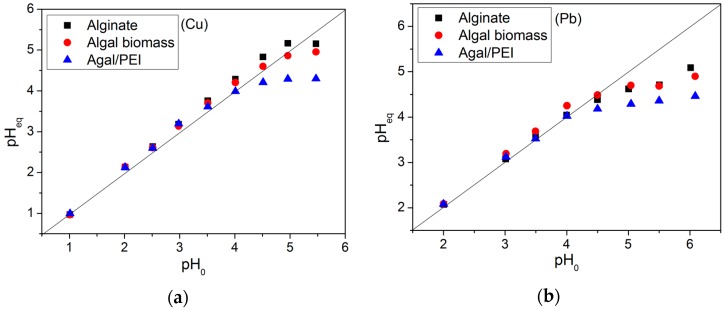
Effect of initial pH on equilibrium pH for (**a**) copper sorption; and (**b**) lead sorption.

Moreover, Figure S2 (which represents the pH variation during metal sorption) indicates that the three sorbents possess a kind of buffering effect: the equilibrium pH in Pb(II) solution only increased from 4.0 to 5.0, 4.3 to 4.9 and 4.0 to 4.5 for alginate, algal and algal/PEI beads, respectively when the initial pH varied from 4.0 to 6.0. This may explain that in Figure 2 the sorption capacities do not significantly increases over initial pH 4. Therefore, for the next part of the study, the sorption experiments were carried out by adjusting the initial pH of the solution to 4. The equilibrium pH tends to decrease when initial pH exceeds pH 4; this is probably due to proton release from the sorbents (deprotonation of carboxylic acid groups; the p*K*_a_s of carboxylic groups in guluronic and mannuronic acid being close to 3.38 and 3.6, respectively, [51]), and the possible formation of hydrolysis products (initially soluble but that can precipitate when exceeding the solubility limit).

### 2.3. Effect of the Composition of the Solution

**Figure 3:**
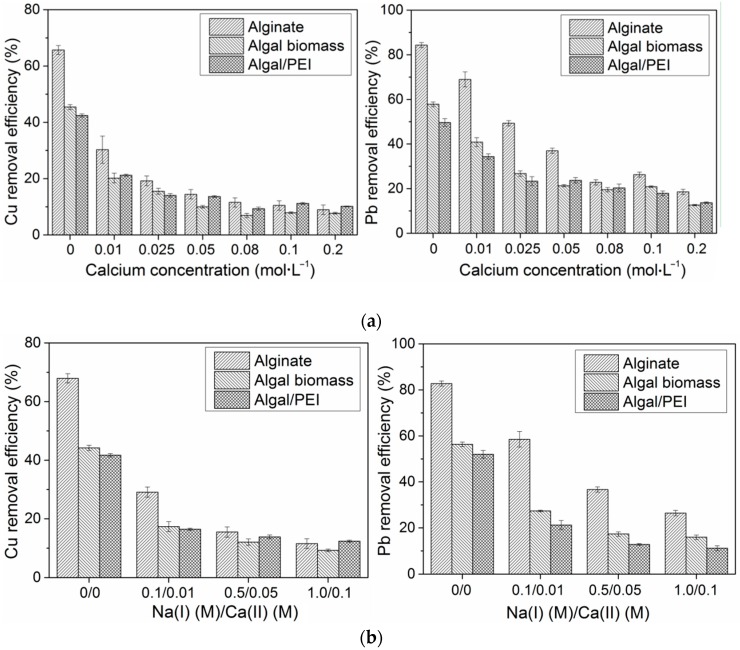
Sorption of Cu(II) and Pb(II) in the presence of salts. (**a**) In the presence of Ca(II); and (**b**) In the presence of Na(I) and Ca(II). The error bars represent the standard deviations (pH_0_ = 4, t = 48 h, T = 25 ± 1 °C; for Cu(II): SD = 400 mg·L^−1^, *C*_0_ = 50 mg·L^−1^; for Pb(II): SD = 200 mg·L^−1^, *C*_0_ = 100 mg·L^−1^).

The sorption of Pb(II) and Cu(II) was tested in the presence of calcium nitrate and sodium nitrate. Previous studies have shown that Na(I) ions have a stronger competitive effect on the sorption of divalent cations by alginate than Ca(II) ions [52]. The stability of the alginate-based sorbents is generally strongly affected by the presence of an excess of sodium ions. Indeed, Na(I) ions are involved in an ion-exchange with Ca(II) ions bound to carboxylic groups on the biopolymer (ionotropic gelation of the biopolymer, Ca-alginate); this ion-exchange leads to the formation of Na-alginate, which turns to be soluble in water. As a consequence, the progressive dissolving of the biopolymer and the disruption of composite capsules may occur. To avoid this phenomenon when investigating the impact of Na(I), a certain amount of Ca(II) (here 10% of Na(I) content) was added to maintain the egg-box structure of the alginate matrix [44]. Figure 3a shows that the presence of Ca(II) significantly inhibited the heavy metal sorption onto all of the sorbents, especially for Cu(II). When the concentration of Ca(II) increased from 0 to 0.01 M, the copper removal efficiency was down by half or more, from 66% to 30% for alginate, 45% to 20% for algal biomass and 42% to 21% for algal/PEI, while for lead, these were only from 84% to 69%, 58% to 41% and 50% to 34%, respectively. The removal efficiency reductions caused by the presence of Na(I) were relatively less (Figure 3b). For example, when the concentration of Na(I)/Ca(II) changed from 0 to 0.1/0.01 M, removal efficiency of Cu(II) decreased from 68% to 29% for alginate beads, from 44% to 17% for algal beads and from 42% to 17% for algal/PEI beads, which were very close to the reduction (36%, 25% and 21% for alginate, algal and algal/PEI beads, respectively) occurring when only Ca(II) was present in the solution. Moreover, when the concentration of Ca(II) was higher than 0.05 M, algal/PEI beads presented a higher removal for Cu(II) than algal beads, suggesting that the incorporation of PEI into algal biomass beads slightly helped in decreasing the inhibiting effect of Ca(II) on Cu(II) removal, while for Pb(II), no improvement by PEI addiction was observed.

These results contrast with the conclusions raised by Bertagnolli et al. [44] for the sorption of a series of metal cations using PEI immobilized in alginate beads: the presence of PEI fraction in the composite sorbent allowed slightly reducing the effect of Ca(II)/Na(I) ions at high concentration of competitor ions. The incorporation of PEI in the composite beads does not appear to improve the sorption performance regarding pH effect (preceding section) nor the selectivity in sorption in the presence of monovalent and divalent earth or alkaline earth metal ions. Therefore, the beneficial effect of the incorporation is mainly limited to the enhancement of sorption properties for other metal ions that are bound through another kind of sorption mechanism. An on-going research performed in parallel to the present study has shown that the incorporation of PEI in algal beads allows substantially increasing the sorption capacity of the material for metal anions such as Pd(II) or Pt(IV) that forms chloro-anions in HCl solutions. Indeed, in this case the composite sorbents can bind tetrachloropalladate anions and hexachloroplatinate anions on protonated amine groups by electrostatic attraction on protonated amine groups of PEI compound (or ion exchange with the counter anion bound to protonated amine groups).

### 2.4. Sorption Isotherms

**Figure 4:**
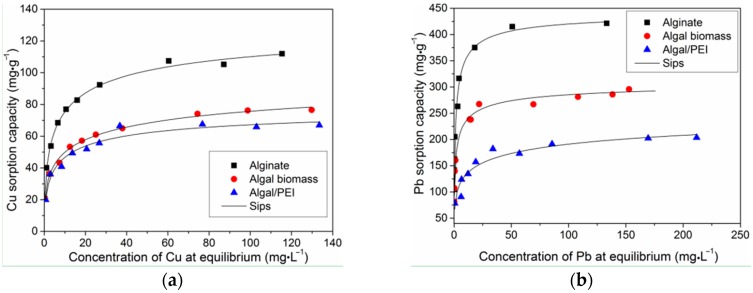
Sorption isotherms of Pb(II) (**a**) and Cu(II) (**b**) (t = 64 h; pH_0_ = 4, T = 25 ± 1 °C; for Cu(II): SD = 400 mg·L^−1^; for Pb(II): SD = 375 mg·L^−1^).

The sorption isotherms represent the distribution of the solute (here Pb(II) and Cu(II)) at equilibrium between the solid phase (the sorbent) and the liquid phase (the solution), when varying metal concentration. This is represented by the plot of *q*_eq_ (sorption capacity at equilibrium) as a function of residual metal concentration: *q*_eq_ = f(*C*_eq_). These curves are characterized by two objective parameters the initial slope of the curve (which is correlated to the affinity of the sorbent for the solute) and the saturation plateau (which is representative of the maximum sorption capacity). The sorption isotherms of Pb(II) and Cu(II) are shown in Figure 4. The curves are all characterized by a common trend: (a) first an initial very steep section corresponding to a strong increase in sorption capacity at low residual metal concentration); and (b) a saturation plateau reached at residual concentrations in the range 50–70 mg metal L^−1^.

The experimental data were fitted by the Langmuir, Freundlich and Sips models, respectively. Parameters were evaluated by *R*^2^ (determination coefficient) and *x*_(10)_^2^ (chi-square). The simulated results and the corresponding parameters are shown in Figure 4 (solid lines) and Table 1, respectively. As is shown, high coefficients and relatively low *x*_(10)_^2^ (*chi-square* test, *p* > 0.1) suggest that Sips model can give a better fit than Langmuir and Freundlich models for both metals. The predicted values of *q*_m_ by Sips model for Pb(II) and Cu(II) were 455 and 138 mg·g^−1^ for alginate, 338 and 117 mg·g^−1^ for algal biomass and 230 and 83 mg·g^−1^ for algal/PEI, respectively. Comparing the predictions from Sips model to the experimental values, Pb(II) could occupy 91 % of the available binding sites in alginate, 87% in algal beads and 69% in algal/PEI beads, while these for Cu(II) are 81%, 66% and 81%, respectively.

Deze et al. [20] also found that less than 100% (83%) of the available binding sites in alginate aerogel was occupied by Cu(II). They suggested that the accessibility or availability of some reactive groups could be limited by steric hindrance. A higher initial concentration leads to more metal ions sorbed and thus, more alginate chains were rearranged. The tighter polymer matrix subsequently hinders further chain rearrangement or makes it more difficult for metal ions to diffuse to the sorption sites and thus results in inaccessibility of some sorption sites. Moreover, *q*_m,exp_ values (in molar units) for Pb(II) are slightly higher (by 13%–19%) than the values for Cu(II) for alginate and algal beads; while for algal/PEI beads the experimental maximum sorption capacity was comparable for Cu(II) and Pb(II) (1.05–1.06 mmol metal g^−1^). This result is in agreement with previous work [53], and three main reasons are generally evocated for explaining differences in the affinities of given metal ions for a sorbent: the ionic radius, the electronegativity and the hydrolysis constant of the metal ions. Hence, comparing Pb(II) over Cu(II) ,it is noteworthy that: (a) Pb(II) has a higher ionic radius than Cu(II) (Pb(II) = 77.5 nm and Cu(II) = 73 nm); (b) Pb(II) has a higher electronegativity of than Cu(II) (Pb(II) = 2.33 and Cu(II) = 1.9), and (3) Pb(II) has a slightly lower p*K*_H_ (negative log of hydrolysis constant) than Cu(II) (i.e., 6.6 for Pb(II) and 6.7 for Cu(II)). In addition, it is generally observed that higher molecular weight metal ions (Pb = 207.2 g·mol^−1^) can be more efficiently removed than lighter metal ions (Cu = 63.55 g·mol^−1^). The maximum sorption capacity observed from experiments (*q*_m,exp_, mmol·g^−1^) of all the sorbents for Pb(II) and Cu(II) followed the order: alginate > algal > algal/PEI. The satisfactorily performance of alginate in sorption ability may result from the abundant carboxylic group, which provided chemical ligands to form metal-ligand complexes. Obviously, the incorporation of PEI does not improve the sorption of Cu(II) or Pb(II). The *q*_m,exp_ of algal/PEI beads decreased by 26% for Pb(II) and 12% for Cu(II) when compared to algal beads. This clearly shows that at least in single-metal solutions, the incorporation of PEI does not bring beneficial effects on the sorption of metal cations (contrary to current results on the sorption of metal anions).

According to the Pearson′s theory the hard acids prefer to associate with hard bases and soft acids prefer to associate with soft bases [54]. Actually, Pb(II) and Cu(II) are generally considered as being part of the borderline class; meaning that they cannot be neither considered as hard nor soft acids, so they do not have special affinity or preference for ligand atoms like O (in carboxylic groups) or N (in amine-type ligands). So the poor differences in their relative affinity for the different sorbents (as appearing in the individual profiles for mono-component solutions) could be anticipated: the softness parameters for Pb(II) and Cu(II) are very close (i.e., 0.41 and 0.38, respectively). On the other hand, the comparison of the affinities coefficients *K*_L_ and *K*_S_ (calculated in molar units) shows a significantly higher affinity of the sorbents for Pb(II) than for Cu(II). Indeed, the *K*_S_ values for Pb(II) vary according: 174, 153 and 70 L·mmol^−1^ for alginate beads, algal beads and algal/PEI beads, respectively. In the case of Cu(II) much lower values were obtained: 24, 19 and 25 L·mmol^−1^ for alginate beads, algal beads and algal/PEI beads, respectively. This means that a certain preference for Pb(II) over Cu(II) could be anticipated for competitive sorption from binary solutions. Complementary experiments are currently performed for investigating the sorption properties of the three sorbents in bi-component solutions. Results demonstrate that the sorption of Pb(II) is less influenced by the presence of Cu(II) than the reciprocal (not shown).

Comparing the sorption capacities of the three sorbents for both Cu(II) and Pb(II) the materials can be classified according the series: alginate beads > algal beads > algal/PEI beads. It is noteworthy that in the case of alginate and algal beads the principal reactive groups are supposed to be the carboxylic functions of guluronic acid and mannuronic acid (which are the constituants of alginate), in addition to other polysaccahrides in the case of algal biomass (including fucoidan, etc.). The alginate beads have sorption capacities that are only 1.4–1.47 times higher than the sorption levels reached with algal beads. The extraction alginate from algal biomass showed a content of about 31% (*w*/*w*). This means that despite a lower amount of alginate in algal biomass the sorption is only increased by 50% for alginate beads while the amount of alginate was multiplied by 3. The presence of other polysaccharides, of other ractive groups (such as proteins), a better dispersion of alginate in the biomass (due to the presence of other constituents, such as cellulose fibers) may contribute to enhance the availability, accessibility or reactivity of functional groups on the polymer chain. Previous studies on silver sorption using chitosan foams reinforced with cellulose fibers [55] have shown that the dispersion of the biopolymer due to the presence of cellulose fibers increased the molar ratio Ag(I)/-NH_2_ at saturation of the material: the dispersion of aminopolysaccharide improved the availability and accessibility of the reactive groups. However, this conclusion should be taken carefully and would probably deserve complementary experimentation since the compositions of alginate and alginate extracted from the *L. digitata* algal biomass used in this study was inversed (See Material and Methods). The affinity coefficients (represented by parameters *K*_L_ and *K*_S_ in the models of Langmuir and Sips) are of the same order of magnitude for the three sorbents in the case of Cu(II) sorption, while for Pb(II) a significant difference was observed between alginate beads and algal beads on one side and algal/PEI beads on the other side (in the case of the composite beads the affinity coefficient was drastically decreased).

The sorption performance of these materials can be compared to other biosorbents. Table S1 (see Supplementary Materials). The diversity in experimental conditions (especially pH) makes sometimes the comparison difficult. However, the order in magnitude for Cu(II) and Pb(II) with the other biosorbents appear to be of the same order of magnitiude than the levels reached with the best materials (i.e., *Laminaria japonica* chemically modified by KMnO_4_ treatment for Pb(II) [56], or *Ulva fasciata* for Cu(II) [57]. The conditioning of the sorbents as spherical beads facilitated the use of the sorbent in fixed-bed columns with enhanced hydrodynamic properties (decrease in head loss, and clogging); this is a substantial benefit compared to the conventional form of algal biomass materials. Compared to alginate beads the cost of the raw material is about 4 times lower (under similar trading conditions) between alginate and algal biomass. In addition, the environmental impact for the elaboration of alginate beads is higher than for the one-pot synthesis of algal-beads since the biopolymer does not need to be extracted and isolated from the raw biomass. Obviously, the sorption capacities are lower than those found for synthetic resins: Kirupha et al. [58] reported sorption capacities as high as 2.1 and 7.7 mmol·g^−1^ for Pb(II) and Cu(II), respectively with poly(2,5-(1,3,4-thiadiazole)benzalimine) resin. Fu et al. [59] reports sorption capacities as high as 3.8 mmol Cu g^−1^ for magnetic poly-acrylic weak acid resin; while Kolodynska et al. [60] highlight the limited effect of pH on the recovery of Cu(II) when using conventional synthetic resins such as Dowex M 4195 and Lewatit MonoPlus TP 220. However, sorbents of biological origin presents an important advantage compared to synthetic materials in terms of life cycle. Indeed, the pyrolisis and incineration of synthetic resins usually produce toxic sub-products (volatile compounds) that may have serious impact on environment [61], compared to the more environmentally friendly degradation of biosorbents.

### 2.5. Uptake Kinetics

**Figure S3:**
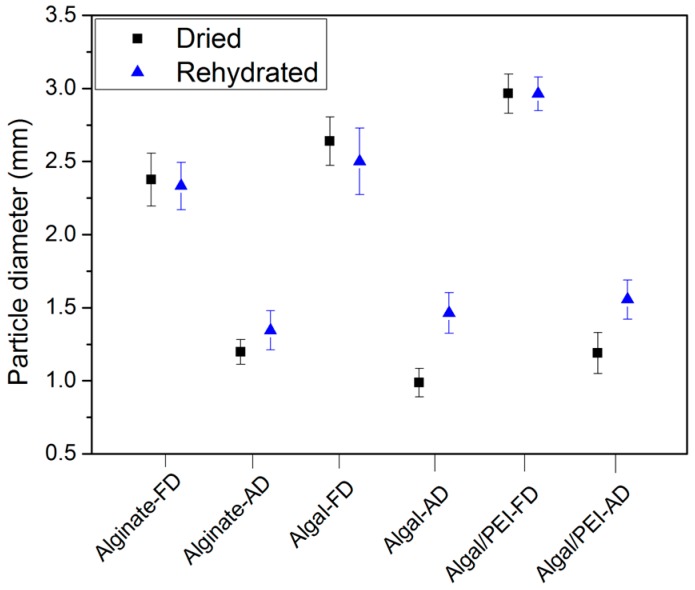
Comparison of diameter between dried sorbents and rehydrated sorbents.

Another important criterion is the velocity of metal binding in the design of a sorption process. Uptake kinetics may be controlled by a series of mechanisms involving the proper reaction rate but also several kinds of mechanisms of resistance to mass transfer (including bulk diffusion, film diffusion and intraparticle diffusion). Providing a sufficient agitation to the reactor allows reducing the impact of resistance to bulk diffusion and to film diffusion and frequently the uptake profiles are modelled using simple equations that jointly or independently take into account the reaction rate (the so-called Lagergren equation for the pseudo-first order rate equation, or the pseudo-second order rate equation) [62] or the resistance to intraparticle diffusion [63]. The hydrogels are subject to strong variations in their diffusion properties when submitted to drying procedures. The uncontrolled drying of hydrogels leads to shrunk structures with depleted performances of mass transfer and it is generally preferable controlling the drying of the hydrogels with a freeze-drying procedure [64] (and even better with drying under supercritical CO_2_ conditions [65]). In order to evaluate the contribution of the resistance to intraparticle diffusion uptake kinetics have been systematically performed with the three sorbents with beads that were submitted to a simple air-drying (AD, so-called xerogels) and to freeze-drying (FD, assimilated to aerogels). The drying step is important for safely storing at long-time the sorbents. In order to minimize the floating step of the beads when immersed in the solution, the beads were rehydrated overnight; this step is not sufficient to restoring the original porous structure of the hydrogel since the drying leads to an irreversible shrinking of the porous structure. However, in the case of freeze-dried materials (FD) the rehydration partially restored the porosity of the beads. This is shown by the comparison of the diameter of the beads before and after rehydration for the different sorbents (Figure S3). The freeze-dried beads were almost unchanged in size after rehydration while a substantial increase of the beads was observed for algal and algal/PEI beads when rehydrated. It is noteworthy that this beneficial effect of the rehydration of the air-dried beads on their size (and then on porosity) cannot be observed in the case of alginate beads. In the case of the pure polysaccharide beads the shrinking is irreversible while algal biomass allows a kind of reversibility: the beads swell and the diameter of the beads substantially increases although much less than with FD beads. The drying strongly impacts the porosity of the beads the composition of algal biomass; different relative fractions of G and M units and the presence of cellulose fibers may explain the higher stability of the beads at drying. In any case, freeze-drying improves the morphological stability of the materials. It is now interesting to verify the impact of this parameter on the kinetic profiles.

**Figure S4:**
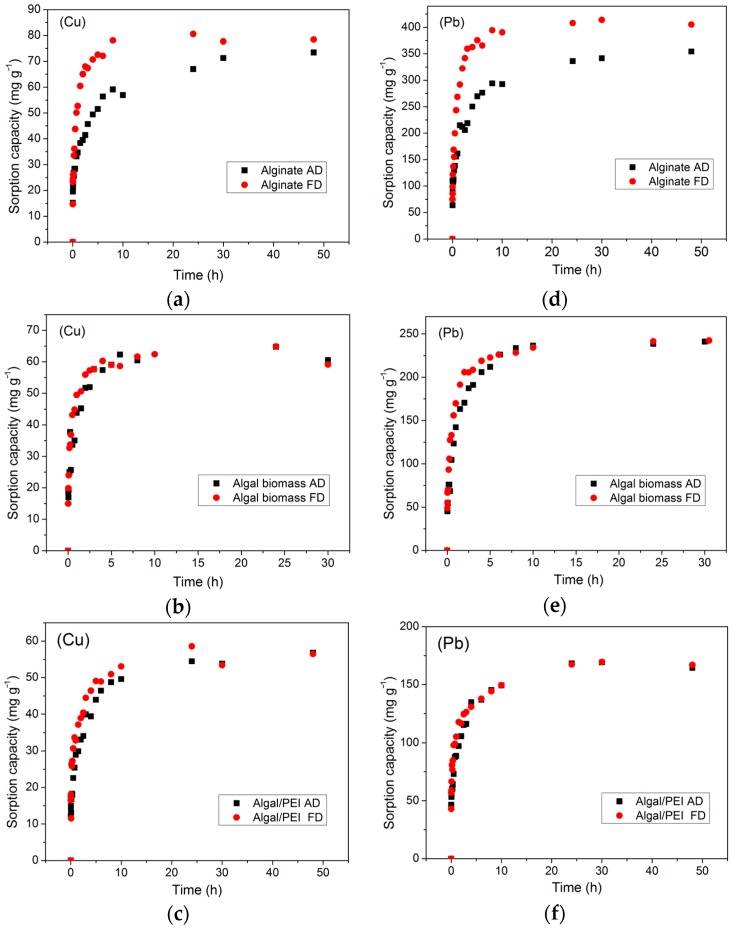
Effect of time on Cu(II) (**a**–**c**); and Pb(II) (**d**–**f**) removal.

Figure S4 presents the uptake kinetics for Cu(II) and Pb(II) sorption using the three sorbents (after AD and FD) by plotting the sorption capacity as a function of time. All the figures are represented by a similar shape: (a) a first initial section with a steep slope, followed by (b) a progressive (and much slower) increase in the sorption capacity, and finally, (c) a saturation plateau (with a very weak slope). In the case of algal beads and algal/PEI beads the kinetic profiles were hardly affected by the mode of drying: the curves almost overlap. A completely different observation can be made for alginate beads: the AD beads were much slower to reach the pseudo-equilibrium (Table 2). The second step (b) represents a much longer step in the process compared to the other materials. In addition, for alginate beads even after 48 h of contact the equilibrium capacities are not exactly equivalent, contrary to the materials elaborated with algal biomass. AD beads can be described as xerogels for which the first rapid phase is related to the sorption in the first external and highly accessible outer layers of the polymer while the more organized and compact structure of the xerogel is less accessible and more resistant to intraparticle diffusion. The rehydration restores at least partially the structure of the algal-based sorbents making the mass transfer of water and of metal ions easier and readily: this contributes to facilitate mass transfer and to enhance uptake kinetics. In the case of algal-based sorbents the SEM photos as well as the study of size particles before and after rehydration confirm the beneficial effects of the presence of cellulose fibers and of the difference in the structure of alginate (inversed G/M fractions, compared to alginate).

Table 2 compares the times of contact required to reach 80% and 99% of total sorption for the different sorbents and with the two modes of drying. To achieve 80% of the equilibrium sorption capacity, it takes about 8 h for air-dried alginate for both Cu(II) and Pb(II) sorption and only 2 h for freeze-dried, while these time gaps were much less between air- and freeze-dried algal-based beads. Moreover, less time was required for Cu(II) removal to reach equilibrium than Pb(II) removal: while 8, 8, and 24 h were sufficient for Cu(II) removal by freeze-fried alginate, algal and algal/PEI, respectively, 24 h was needed for Pb(II) removal for all sorbents. The difference in equilibrium time for Cu(II) and Cd(II) sorption using alginate aerogels was attributed to effects of steric hindrance [20]. The sorption of metal ions induces a rearrangement of alginate chains due to coordination with metal ions. Therefore, more metal ions sorbed onto the sorbents at a longer time will lead the porous matrix to a tighter polymer network that possibly hinders further chain rearrangement or makes it more difficult for metal ions to diffuse to the sorption sites, resulting in a higher equilibrium time. Due to a higher affinity of the sorbents for Pb(II) than Cu(II), the hindering effect is expected to be more significant. This could explain the little differences observed in equilibrium times. It is noteworthy that at long contact time (i.e., higher than 24 h) a slight increase in the residual concentration of copper in the solution was observed, probably due to a partial degradation of the alginate capsules under strong agitation. This was not observed for Pb(II) sorption and for other sorbents.

**Figure 5:**
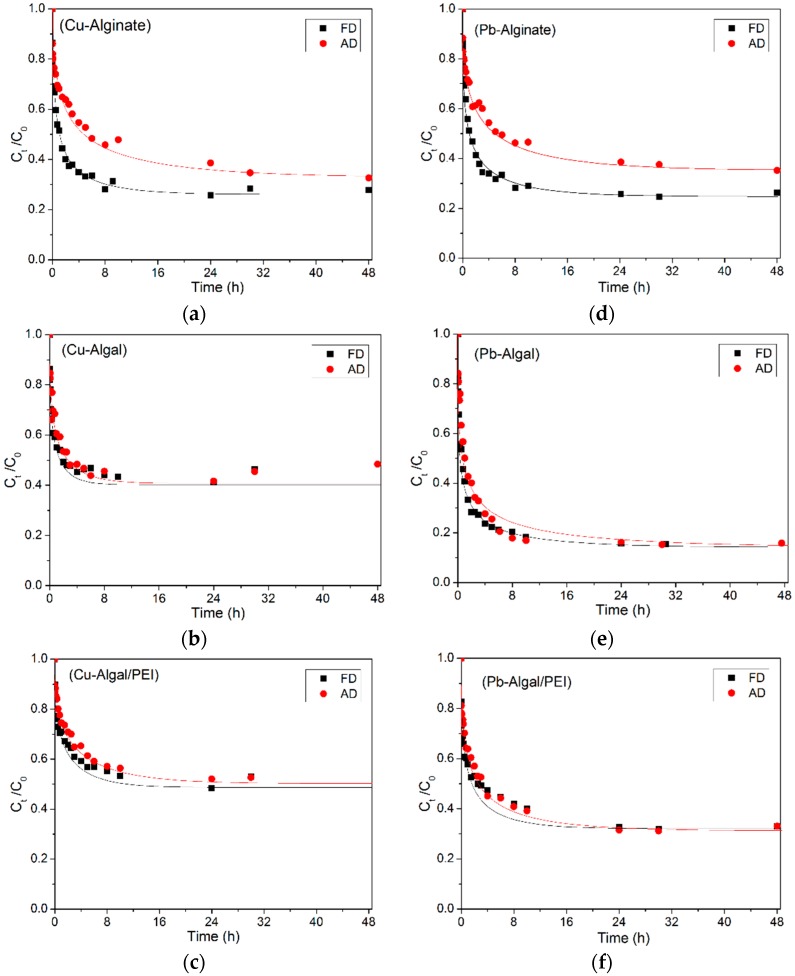
Modeling of uptake kinetics with the Crank Equation ((**a**–**c**) for Cu(II) removal, (**d**–**f**) for Pb(II) removal) (pH_0_ = 4, T = 25 ± 1 °C; for Cu(II): SD = 400 mg·L^−1^, *C*_0_ = 50 mg·L^−1^; for Pb(II): *C*_0_ = 100 mg·L^−1^, SD = 200 mg·L^−1^, 375 mg·L^−1^ and 400 mg·L^−1^ for alginate, algal and algal/PEI beads, respectively).

The pseudo-second order rate equation and the pseudo-first order rate equation [64] were tested for fitting kinetic profiles: these models failed to fit the uptake kinetics (not shown) and the Crank equation (resistance to intraparticle diffusion, Equation (6)) revealed much more efficient for modeling the experimental profiles. The Figure 5 shows the kinetic modeling of Pb(II) and Cu(II) sorption with the Crank equation. The intraparticle diffusion coefficients (*D*_e_) for freeze-dried and air-dried sorbents are summarized in Table 3: the *D*_e_ values were systematically higher for freeze-dried materials than for air-dried materials, regardless of the metal. The freeze-drying process improves the diffusion properties of all the sorbents. Moreover, no obvious trend was found between *D*_e_ for Cu(II) and Pb(II) uptakes. The values of the diffusivity coefficient were found several orders of magnitude lower than the self-diffusivity of Cu(II) (i.e., 4.28 × 10^−8^ m^2^·min^−1^) and Pb(II) (i.e., 5.67 × 10^−8^ m^2^·min^−1^) in water [66]. This confirms that the resistance to intraparticle diffusion plays a significant role in the control of uptake kinetics. These observations on diffusivity coefficients are consistent with the SEM observations and the changes in the size of sorbent beads (and then on their porous network).

## 3. Materials and Methods

### 3.1. Materials

Alginate (Protanal 200S) was supplied by FMC (La Madeleine, France). Brown algal biomass (*Laminaria digitata*) was purchased from Setalg (Pleubian, France). It was washed, dried at 50 °C overnight and grinded (size fraction: <250 µm). The procedure described by McHugh [67] and slightly modified by Bertagnolli et al. [68] was used for quantifying the amount of alginate in *L. digitata*: (i.e., 31%, *w*/*w*). The fractions of mannuronic and guluronic acids (M/G) in alginate and alginate extracted from algal biomass were determined by NMR analysis [69]: 0.37/0.63 and 0.62/0.38, respectively.

### 3.2. Reagents

The Cu(II) and Pb(II) solutions were prepared using analytical grade Cu(NO_3_)_2_ and Pb(NO_3_)_2_ (Sigma-Aldrich, Taufkirchen, Germany) dissolved in deionized water. Polyethylenimine (low molecular weight of 600–800, water free) was obtained from Sigma-Aldrich (Saint-Louis, MO, USA). Calcium chloride (>99.5%) and formic acid (>99%) were purchased from Chem-Lab (Chem-Lab NV, Zedelgem, Belgium). Calcium carbonate and glutaraldehyde (50 wt %) were purchased from Sigma-Aldrich. All other reagents used were of analytical grade.

### 3.3. Methods

#### 3.3.1. Preparation of Sorbents

For alginate beads, 8 g of alginate were mixed with 4 g of CaCO_3_ into 388 mL of Milli-Q water. The mixture was marked as A.

For algal beads, 4 g of algal biomass were mixed with 8 g of Na_2_CO_3_ into 1200 mL of demineralized water. The mixture was maintained at 50 °C for 24 h and then mixed with 8 g of CaCO_3_. After that, the mixture was divided into 2 equal parts. One part was used for manufacturing algal/PEI beads, while the other part was marked as B.

For Algal/PEI beads, 45 g of PEI were added into 500 mL Milli-Q water with gentle agitation. Then, the solution was mixed with 45 mL of glutaraldehyde (50 wt %) under fast agitation. After this step, the mixture was maintained for 24 h at room temperature. Then, it was washed twice with 4 L of demineralized water, filtrated and freeze-dried (−52 °C, 0.1 mbar). Thereafter 2 g of the mixture, grinded and sieved through 200 mesh sieve, were added into the as-prepared algal mixture. The mixture was marked as C.

Then the mixture A, B and C were separately distributed dropwise into a solution containing both CaCl_2_ (1%, *w*/*w*) and formic acid, CH_2_O_2_ (1%, *v*/*w*). After keeping the gel beads in the CaCl_2_/CH_2_O_2_ solution under agitation for 24 h, the beads were washed twice and maintained in 1 L of demineralized water. Then the solution pH was adjusted to 4 using 0.1 M NaOH. Finally, those beads were rinsed with pure water and then divided into 2 parts. One was air-dried (25 ± 1 °C, two days). The other one was freeze-dried (−52 °C, 0.1 mbar, two days).

#### 3.3.2. Metal Solutions and Measurement

Stock solutions (1 g·L^−1^) of Pb(II) and Cu(II) were prepared by dissolving the exact quantity of Pb(NO_3_)_2_ and Cu(NO_3_)_2_ in Milli-Q water. Working solutions were obtained by diluting the stock solutions to the desired concentrations. Before the sorption process, the pH of each test solution was adjusted to the required value by using 0.1 M HNO_3_ or NaOH. The final pH was determined in order to evaluate the potential precipitation phenomena associated with pH variation. Initial and equilibrium metal concentrations were subsequently determined using an inductively coupled plasma atomic emission spectrometer ICP-AES ACTIVA M (HORIBA JOBIN YVON, Longjumeau, France) after samples were filtered.

### 3.4. Sorption Process

#### 3.4.1. Effect of pH

The sorption experiments were conducted within the initial pH range of 1–5.5 for Cu(II) (sorbent dosage of 0.2 g·L^−1^ and contact time of 48 h) and initial pH range of 2–6 for Pb(II) (dosage of 0.17 g·L^−1^ (alginate beads) and 0.38 g·L^−1^ (algal beads and algal/PEI beads) and contact time of 48 h). The pH of the solution was adjusted using 0.1 M HNO_3_ or NaOH. The initial and final values of pH were measured using a pH-meter cyber scan pH 6000 (Eutech instruments, Nijkerk, The Netherlands).

#### 3.4.2. Effect of Coexisting Ions

The influence of Na(I) and Ca(II) was evaluated by sorption experiments in the presence of Ca(NO_3_)_2_ (0–0.2 M) or NaNO_3_/Ca(NO_3_)_2_ (concentration of Na(I)/Ca(II) changed from 0 to 1/0.1 M) with the sorbent dosage of 0.2 and 0.4 g·L^−1^ for Pb(II) and Cu(II), respectively, pH of 4 and contact time of 48 h. When investigating the effect of Na(I) calcium ions were added to prevent the degradation of the beads; indeed, an excess of Na(I) displaces, by ion-exchange, Ca(II) from ionotropically gelled beads with loss of stability of the material.

#### 3.4.3. Sorption Isotherm

The sorption isotherms were investigated at pH 4 with metal concentration ranging from 10 to 200 mg·L^−1^ for Cu(II) and 30 to 300 mg·L^−1^ for Pb(II). Three sorption isotherm equations were used to analyze the sorption data, which are namely: Langmuir, Freundlich and Sips isotherms and they can be expressed by Equations (3)–(5), respectively.

(3) $$q_{e}=\frac{q_{m}K_{L}C_{e}}{1+K_{L}C_{e}}$$

(4) $$q_{e}=K_{F}C_{e}^{1/n_{F}}$$

(5) $$q_{e}=\frac{q_{m}K_{S}C_{e}^{1/n_{s}}}{1+K_{S}C_{e}^{1/n_{s}}}$$

where *q*_e_ (mg·g^−1^) is the equilibrium amount of metal sorbed per unit weight of adsorbent; *C*_e_ (mg·L^−1^) is the equilibrium concentration of metal in aqueous solution; *q*_m_ (mg·g^−1^) is the maximum sorption capacity of the sorbent at saturation of the monolayer; *K*_L_ (L·mg^−1^) is the Langmuir equilibrium coefficient; *K*_F_ (mg·g^−1^) and *n_F_* are the Freundlich constants, which are indicators of the capacity and intensity of sorption, respectively; *K*_S_ (L·mg^−1^) and *n*_s_ are the constants of Sips model. Values for 1/*n*_s_ close to zero are generally associated to heterogeneous sorbents, while values closer to 1.0 correspond to sorbent with relatively homogenous binding sites. The parameters of the models were determined using the DataFit software (Oakdale Engineering, v. 9.0, Oakdale, PA, USA).

#### 3.4.4. Kinetic Study

For kinetic studies, air dried and freeze dried sorbents were compared. However, the beads were soaked into water overnight prior to kinetic tests: a small amount of water was dropped onto the beads to moisten and limit the lag phase associated to re-hydration and to limit the flotation of the beads when dropped in the metal ion solution. For Pb(II), 0.2 g of alginate, 0.375 g of algal beads or 0.4 g of algal/PEI beads were agitated with 1 L of metal solution (*C*_0_ = 100 mg·L^−1^; pH_0_ 4; agitation speed, *v* = 150 rpm; Temperature, *T* = 25 ± 1 °C). For Cu(II), 0.4 g of sorbent beads were shaken with 1 L of metal solution (*C*_0_ = 50 mg·L^−1^; pH_0_ 4; *v* = 150 rpm; *T* = 25 ± 1 °C). Four milliliters of sample were taken out from the solution at desired time intervals over a total period of 2880 min (i.e., 48 h). The intraparticle diffusion coefficient (*D*_e_, effective diffusivity, m^2^·min^−1^) was determined using the Crank equation (Equation (6)), assuming the solid to be initially free of metal, and the kinetics to be only controlled by intraparticle diffusion resistance:

(6) $$\frac{q_{t}}{q_{\mathrm{eq}}}=1-\sum_{n=1}^{\infty }\frac{6\alpha (\alpha +1)exp(\frac{-D_{e}q_{n}{}^{2}t}{r^{2}})}{9+9+q_{n}{}^{2}\alpha^{2}}$$

where *q*_t_ and *q*_eq_ are the metal concentrations in the beads at time t and equilibrium, respectively; *r* is the radius of the sorbent and *q*_n_ values are the non-zero roots of the Equation (7):

(7) $$\tan q_{n}=\frac{3q_{n}}{3+\alpha q_{n}{}^{2}}$$

with

(8) $$\frac{mq_{\mathrm{eq}}}{VC_{o}}=\frac{1}{1+\alpha }$$

where *m* is the mass of sorbent added into the solution. The Mathematica software (Wolfram, Paris, France) was used to calculate the intraparticle diffusion coefficient, *D*_e_, and to simulate experimental data (using an in-lab proprietary calculation module).

### 3.5. Statistic Methods 

Sorption capacities observed from experiments were compared with those calculated from different models by using *chi-square* test (*x*^2^). *x*^2^ was calculated as follows:

(9) $$\chi^{2}=\sum_{i=1}^{n}\frac{{\left(q_{c}-q_{t}\right)}^{2}}{q_{t}}$$

where *q_t_* is observed value and *q*_c_ is calculated value.

## 4. Conclusions and Perspectives

In this work, alginate and algal biomass (*L. digitata*) were shaped under the form of spherical beads by homogeneous Ca ionotropic gelation. This method, alternative to the conventional external ionotropic gelation method, allows synthesizing stable and homogeneously porous materials with highly porous properties. The method was applied on alginate as the reference material but also for algal biomass (with and without incorporation of glutaraldehyde-crosslinked PEI). The drying method has a dramatically impact on alginate beads (the collapse of the porous network is irreversible in the case of air-drying compared to freeze-drying) while in the case of algal biomass beads the method of drying has a weaker effect: after rehydration the beads regain a part of their size and porosity (partial swelling) for air-dried materials and completely for freeze-dried materials. The presence of cellulose fibers in the algal biomass is expected to improve this beneficial effect. Then algal biomass offers more flexibility in the management of drying process compared to alginate beads.

Batch sorption experiments show that algal beads present good sorption performance for both Cu(II) and Pb(II), which could be explained by the fact that the algal biomass (*L. digitata*) used in this study contains alginate with a high ratio of mannuronic acid to guluronic acid (M/G ratios) [53] and an increase in M/G ratios of alginate generally lead to increasing affinity for divalent cations [70]. Although the maximum sorption capacities observed from experiments of pure alginate beads (415 mg·g^−1^ for Pb(II) and 112 mg·g^−1^ for Cu(II)) are higher than algal beads (296 mg·g^−1^ for Pb(II) and 77 mg·g^−1^ for Cu(II)) (Table 1), it is noteworthy that the alginate extraction yield from brown algal was only around 31% and the manpower and time required during the extraction process make this resource much more expensive and less environmentally friendly than algal biomass. Moreover, the less usable residue remained after alginate extraction may bring undesirable problems to solid waste management or may cause secondary pollution associated with its detritus. In addition, a previous study [53] shows that the sorption capacities of this algal biomass (*L. digitata*) encapsulated in alginate beads by adding an extra alginate resource were 87 mg·g^−1^ for Cu(II) and 372 for Pb(II) under an initial solution pH of 4.5, while for the beads without adding extra alginate in this study, these values were 77 and 296 mg·g^−1^, respectively under a lower initial pH condition (pH = 4), again revealing that algal beads obtained by one-pot synthesis process not only reduce the preparation cost but also present comparable sorption capacities for Cu(II) and Pb(II). The incorporation of PEI derivatives into algal beads slightly decreased the sorption properties for Cu(II) and Pb(II), meaning that the presence of amine groups provided by the PEI derivatives does not enhance the sorption of these two metal cations. The result of effect of drying process on uptake kinetics show that, in the case of alginate, a strict control of the drying process is required for maintaining the porous characteristics of the hydrogel to get a fast removal of Cu(II) and Pb(II), while in the case of algal-based sorbents the swelling of the material restores the porous characteristics of the original hydrogel and minimizes the impact of the drying process. The composition of the algal biomass (including cellulose-like fibers) is expected to improve these swelling and porous properties.

In conclusion, a low-cost and green sorbent, algal biomass beads, was prepared by one pot synthesis process, during which, alginate is simultaneously extracted and used for shaping/conditioning the sorbent as spherical beads. Experimental results demonstrate the high efficiency of this sorbent for Cu(II) and Pb(II) removal. In addition, the swelling process after air-drying is sufficient to maintain its porous characteristic, requiring no strict drying process (freeze-drying). The results of this research have important significance on preparation of algal biomass-based beads by one-pot synthesis process as an efficient heavy metal sorbent. The conditioning of the algal biomass under the form of beads allows using these materials in fixed-bed column while minimizing the risk of clogging and head loss pressure, contrary to the conventional ribbon-like shape of algal biomass.

Metal desorption and sorbent recycling were not tested in the present study. However, recent results have shown that slightly acidic solutions (i.e., HCl solutions at pH 2) can readily desorb Zn(II), Cu(II) and Cd(II) from metal-loaded Alginate/PEI beads [44]. Similar trends are expected for the desorption of Cu(II) and Pb(II) from the present sorbents. It is noteworthy that the presence of small amounts of calcium chloride (at concentration close to 0.05 M) in the eluent may contribute to reinforce the stability of the sorbents.

Further studies will investigate the possibility to use this type of material as support for catalytic metals (heterogeneous catalysis) by binding metal ions such as Pd(II). Post-reduction (with chemical agent) will help in designing green supported catalysts. On-going research has shown that these materials can bind small amounts of precious metals while the incorporation of PEI (cross-linked with glutaraldehyde) drastically increases the sorption properties of the composite. The protonated amine groups of PEI are highly reactive for binding tetrachloropalladate anions through anion-exchange/electrostatic attraction. This property gives sense to incorporating PEI in algal beads for the recovery of metal anions in slightly acidic conditions.

## Figures and Tables

**Table 1:** Isotherm constants for the sorption of Cu(II) and Pb(II) onto the three sorbents.

Models	Parameters	Alginate		Algal Biomass		Algal/PEI	
		Pb(II)	Cu(II)	Pb(II)	Cu(II)	Pb(II)	Cu(II)
	*q*_m,exp_	414.8	111.9	295.8	76.5	217.5	67.5
	*q*_m,exp_^(a)^	2.00	1.76	1.43	1.20	1.05	1.06
Langmuir	*q*_m_	390.3	107.5	264.2	74.5	201.4	66.3
	*K*_L_	1.69	0.32	1.90	0.26	0.20	0.31
	*R*^2^	0.94	0.93	0.85	0.87	0.71	0.88
	*x*_(10)_^2^	35.4	14.3	12.9	12.9	5.3	7.4
Freundlich	*K*_F_	195.9	43.1	144.3	30.4	89.5	28.9
	*n*_F_	5.78	4.71	6.63	4.96	6.06	5.31
	*R*^2^	0.88	0.95	0.88	0.97	0.88	0.90
	*x*_(10)_^2^	61.7	8.4	15.4	1.9	6.1	4.8
Sips	*q*_m_	454.8	138.4	337.8	116.7	315.9	82.9
	*K*_S_	0.84	0.37	0.74	0.30	0.34	0.39
	*n*_S_	1.60	1.95	2.27	2.55	3.04	1.91
	*R*^2^	0.98	0.99	0.96	0.99	0.91	0.95
	*x*_(10)_^2^	12.0	0.3	3.9	0.4	4.3	1.6

*q*_m_ and *K*_F_: mg metal·g^−1^; *K*_L_ and *K*_S_: L·mg^−1^; *n*_F_ and *n*_S_: dimensionless; ^(a)^ molar units.

**Table 2:** Contact time for reaching 80% or 99% of equilibrium sorption capacity for freeze- and air-dried sorbents.

Percent (%)	Metal	Contact Time (h)					
		Alginate Beads		Algal Beads		Algal/PEI Beads	
		AD	FD	AD	FD	AD	FD
80	Cu	8	2	1.5	0.5	5	3
	Pb	8	2	3	1.5	4	4
99	Cu	48	8	8	8	48	24
	Pb	30	24	24	24	24	24

**Table 3:** Modeling of sorption kinetics of Pb(II), Cu(II) using alginate, algal biomass and algal/PEI Beads (Crank Equation).

Metal	*D*_e_ × 10^−^^11^ (m^2^·min^−^^1^)					
	Alginate Beads		Algal Beads		Algal/PEI Beads	
	AD	FD	AD	FD	AD	FD
Cu	3.5	22.4	1.8	8.8	1.0	6.1
Pb	4.3	17.6	2.4	13.5	0.95	4.4

## Supplementary Materials

**Table S1:** Comparison of Cu(II) and Pb(II) sorption properties for a series of biosorbents.

**Chitosan-Based Sorbents**							
**Sorbents**	***q*_m_ for Cu(II)**	***q*_m_ for Pb(II)**	**pH**		**Sorbent Particle Size**		**References**
Chitosan-tripolyphosphate beads	0.43 ^a^	0.28 ^a^	4.5		<200 μm		[71]
Crosslinked chitosan	2.06 ^a^	0.81 ^a^	6 for Cu(II); 5 for Pb(II)		100–150 μm		[72]
Chitosan-coated sand	0.19 ^b^	0.11 ^b^	–		0.50 mm ^d^		[73]
Cross-linked metal-imprinted Chitosan	0.31 ^a^	0.36 ^a^	5		250–500 μm		[74]
**Agriculture Wastes**							
**Sorbents**	***q*_m_ for Cu(II)**	***q*_m_ for Pb(II)**	**pH**		**Sorbent Particle Size**		**References**
*Rosa bourbonia* petals	2.35 ^a^	0.69 ^a^	5 for Cu(II); 4.5 for Pb(II)		0.25 mm ^d^		[75]
Chemically modified orange peel	0.24 ^a^	0.35 ^a^	5		0.2mm ^d^		[76]
Cabbage waste	0.20 ^b^	0.30 ^b^	6		75–300 μm		[77]
**Non-modified Algal Biomass**							
**Sorbents**	***q*_m_ for Cu(II)**	***q_m _* for Pb(II)**	**pH**		**Sorbent Particle Size**		**References**
*Posidonia oceanica*	–	0.53 ^a^	5		<125 μm		[78]
*Padina* sp.	1.14 ^a^	1.25 ^a^	5		0.5–0.8 mm		[14]
*Caulerpa lentillifera*	0.67 ^a^	0.12 ^a^	4		0.9 mm ^d^		[12]
*Ulva fasciata*	1.57 ^a^	–	5.5		0.5 mm ^d^		[57]
*Spirogyra* sp.	0.61 ^a^	0.44 ^a^	5		150–250 mesh		[79]
*Gelidium* sp.	0.52 ^a^	–	5.3		0.25–1 mm		[17]
*U. lactuca*	–	0.61 ^b^	4.5		–		[11]
*Fucus vesiculosus*	1.66 ^a^	1.02 ^a^	5		<0.5 mm		[16]
*G. oblongata*		0.43 ^b^	5		500–850 mm		[10]
*Padina* sp.	1.14 ^a^	1.25 ^a^	5		0.5–0.8 mm		[14]
*Kappaphycus alvarezii*	0.47 ^a^	0.51 ^a^	4.5		0.75 mm ^d^		[80]
**Modified Algal Biomass**							
**Sorbents**	***q*_m_ for Cu(II)**	***q*_m_ for Pb(II)**	**pH**	**Treatment**		**Sorbent Particle Size**	**References**
*Laminaria japonica*	–	1.52 ^c^	5.3	KMnO_4_		0.3–0.45 mm	[56]
*Cystoseira barbata*	–	1.10 ^a^	4	CaCl_2_		0.6–1.0 mm	[81]
Green algae (unspecified species)	–	0.48 ^a^	5	Dimethyl sulfoxide (DMSO)		<0.75 μm	[82]
*Ulva lactuca*	0.16 ^a^	0.23 ^a^	5.5	Immobilization into agar beads		–	[83]
Algal biomass (*Fucus vesiculosus*) beads	0.62 ^a^	2.3 ^a^	5	Alginate ^e^		–	[84]
Alginate beads	1.76 ^b^	2.00 ^b^	4	Homogeneous ionotropic gelation ^f^		2.4 mm ^d^	This study
Algal biomass (*Laminaria digitata*) beads	1.20 ^b^	1.43 ^b^	4	Idem ^g^		2.6 mm ^d^	This study
Algal/PEI beads	1.06 ^b^	1.05 ^b^	4	Idem ^g^		3.0 mm ^d^	This study

^a^ *q*_m_ from Langmuir model (mmol·g^−1^); ^b^ *q*_m_ from experiments (mmol·g^−1^); ^c^ *q*_m_ from Langmuir-Freundlich (or Sips) model (mmol^−1^); ^d^ average particle diameters; ^e^ immobilization in alginate beads with heterogeneous Ca(II) ionotropic gelation; ^f^ heterogeneous ionotropic gelation; ^g^ heterogeneous ionotropic gelation, without addition of alginate.
